# Immune Suppression Mediated by STAT4 Deficiency Promotes Lymphatic Metastasis in HNSCC

**DOI:** 10.3389/fimmu.2019.03095

**Published:** 2020-01-15

**Authors:** Kelvin Anderson, Nathan Ryan, Greta Volpedo, Sanjay Varikuti, Abhay R. Satoskar, Steve Oghumu

**Affiliations:** ^1^Department of Pathology, The Ohio State University Wexner Medical Center, Columbus, OH, United States; ^2^Department of Microbiology, The Ohio State University, Columbus, OH, United States

**Keywords:** squamous, carcinoma, metastasis, myeloid, suppressor

## Abstract

Head and neck squamous cell carcinoma (HNSCC) is a prevalent form of cancer with 5-years survival rates around 57%, and metastasis is a leading cause of mortality. Host-derived immunological factors that affect HNSCC tumor development and metastasis are not completely understood. We investigated the role of host-derived signal transducer and activator of transcription 4 (STAT4) during experimental HNSCC using an aggressive and metastatic HNSCC cell line, LY2, which was orthotopically injected into the buccal sulcus of wild type (WT) and STAT4 deficient (*Stat4*^−/−^) BALB/c mice. Necropsies performed at terminal sacrifice revealed that *Stat4*^−/−^ mice displayed comparable primary tumor growth to the WT mice. However, the rate and extent of lymph node and lung metastasis among *Stat4*^−/−^ mice was significantly higher. Downstream analyses performed on primary tumors, draining lymph nodes, spleens and bone marrow revealed significant upregulation of lymphocytic immunosuppressive biomarkers as well as an accumulation of granulocytic MDSC subpopulations in draining lymph nodes of metastatic *Stat4*^−/−^ mice. Further, we observed a significant decrease in T_H_1, T_H_17, and cytotoxic activity in tumor bearing *Stat4*^−/−^ compared to WT mice. Our results demonstrate that STAT4 mediates resistance to HNSCC metastasis, and activation of STAT4 could potentially mitigate lymphatic metastasis in HNSCC patients.

## Introduction

Head and neck squamous cell carcinoma (HNSCC) is one of the most common forms of cancer, making up about 90% of oral cancer cases. In 2020, there are expected to be roughly 833,000 new cases of HNSCC worldwide (1), 53,000 of which will occur in the United States (2). Five-year survival rates for HNSCC have remained at around 57%, with HNSCC-associated mortality partly attributable to complications with metastasis. Indeed, untreated metastatic patients are expected to live an average of 4 months (3). It is therefore imperative that factors governing tumor metastasis in HNSCC be fully characterized.

Cancer metastasis is a complex process, involving the dissemination of cancer cells from the primary tumor site to distal organs. The sentinel lymph nodes are known to be the secondary sites to which tumors initially spread (4, 5), and lymph node metastasis has been shown to correlate with distal metastases, which affects tumor progression and prognosis (6, 7). Sentinel lymph node metastasis result from a series of molecular and cellular changes that facilitate the entry, survival and proliferation of the tumor cell in the lymph node. Although the mechanisms of lymph node metastasis are under active investigation, the immunological factors that govern tumor cell entry and proliferation in the lymph node as well as subsequent metastasis to distal organs in HNSCC are not completely understood.

Signal transducer and activator of transcription (STAT) 4 is a transcription factor that has been implicated in carcinogenesis and tumor progression (8). Phosphorylation of STAT4 by the signaling tyrosine kinases JAK2 and TYK2 occurs in response to receptor stimulation by interleukin (IL) 12, (9), or IL-23 (10). Following STAT4 homo-dimerization in response to IL-12 or hetero-dimerization with STAT3 in response to IL-23, nuclear translocation initiates transcription of genes associated with upregulation of NK cell cytotoxicity (11), differentiation of CD4+ T_H_1 immunity (12, 13), and stimulation of IFN-γ production in macrophages, dendritic cells, CD4^+^, CD8^+^, and NK cells (14, 15).

Given its significant immunomodulatory functions, the role of STAT4 in carcinogenesis has been extensively studied. Previous reports demonstrate that decreased levels of STAT4 as indicative of worse prognoses in hepatocellular carcinoma (16), while high STAT4 expression has been linked to reduced tumor recurrence (17), and improved stage 3 prognosis in patients with gastric cancer (18). Similarly, STAT4 expression has been linked to improved survival in breast and late-stage ovarian cancer (19, 20), while decreased levels of phosphorylated STAT4 in peripheral blood mononuclear cells is associated with metastasis in melanoma patients (21, 22). Interestingly, a contrasting study showed a direct relationship between STAT4 expression and tumor development in ovarian cancer, where its overexpression was associated with epithelial-to-mesenchymal transition of cancer cells, resulting in metastasis (23). Another study linked STAT4 to tumor growth and invasion in colorectal cancer (24).

Up to this point, the functions of STAT4 in carcinogenesis and tumor metastasis are paradoxical, appearing to be dependent on the cancer type. Further, there is extremely limited knowledge on the role of STAT4 during HNSCC development and metastasis. In this study, we investigated the effect of host STAT4 deficiency on HNSCC tumor development and metastasis, using an experimental orthotopic model of HNSCC in immunocompetent BALB/c mice and an aggressive, metastatic murine oral cancer cell line, LY2. This model provides a suitable syngeneic *in-vivo* system to examine the role of immunological mediators during HNSCC in immunocompetent mice. Our results demonstrate that STAT4 deficiency diminishes anti-tumor immune responses and promotes accumulation of immunosuppressive myeloid cell populations to promote HNSCC metastasis.

## Materials and Methods

### Tumor Cell Lines

Murine LY2 metastatic HNSCC cell line, a generous gift from Dr. Nadarajah Vigneswaran were derived from PAM212 squamous cell carcinoma cells that developed lymph node metastasis after injection into BALB/C mice (25). Cells were cultured in advanced DMEM/F12 media (Life Technologies, Waltham, MA, USA), supplemented with 2% fetal bovine serum (Corning, Corning, NY, USA), 100 μg/mL penicillin G, 100 μg/mL streptomycin, and 2 mM L-glutamine (Life Technologies) at 37°C and 5% CO_2_. Cells were grown to 75% confluence and harvested by trypsinization. 5.0 × 10^5^ cells in 20 μl media were mixed 1:1 with Matrigel (Corning) and injected into the right buccal mucosa of experimental BALB/c mice.

### Animals

Wild type (WT) and STAT 4 deficient (*Stat4*^−/−^*)* BALB/c mice, age matched at ~8 weeks, were used for these studies. Experimental WT and *Stat4*^−/−^ mice (*n* = 5 per group) were injected with LY2 HNSCC cells while control WT or *Stat4*^−/−^ BALB/c mice (*n* = 4 per group) were not injected with LY2 cells. WT mice were acquired from Jackson Laboratories (Bar Harbor, ME, USA) and *Stat4*^−/−^ mice were obtained as described previously (13). Animals were housed in Ohio State University animal facilities in accordance with state and federal guidelines provided by University Laboratory Animal Resources (ULAR). Experiments involving the use of animals were approved by the Institutional Animal Care and Use Committee (Protocol #2018A00000054) and Institutional Biosafety Committee (IBC) of the Ohio State University.

### Analysis of Tumor Progression and Metastasis

WT (*n* = 5) or *Stat4*^−/−^ (*n* = 5) BALB/c mice were injected with LY2 cells in the right buccal mucosa. Weights and tumor volumes from each mouse were taken twice weekly until sacrifice at day 50 post tumor injection. Tumor measurements were acquired using electronic calipers, and tumor volumes were calculated using the equation V = (L^*^W^2^)/2mm^3^; where L = longest tumor diameter, W = shortest diameter of tumor. One *Stat4*^−/−^ mouse was removed at day 42 due to advanced illness. At terminal sacrifice, primary tumors, bone marrow, cervical lymph nodes, and spleens of each mouse were harvested. Tumors were weighed and analyzed by RT-qPCR and flow cytometry. Lungs were also collected and placed in 3 mL Bouin's solution (MilliporeSigma, Burlington, MA, USA) for analysis of lung metastasis. Lymph node metastasis was determined by gross visualization and histologic analysis of hyperplastic tumor cells within the lymph nodes. Lung metastasis was determined by gross inspection for metastatic nodules and confirmed histologically in H&E stained slides by a certified pathologist.

### Flow Cytometry

Single cell suspensions from primary tumors, cervical lymph nodes, spleens and bone marrow were generated and passed through a 70 μm nylon mesh. Spleen samples were pre-treated with ACK lysis buffer to remove erythrocytes. Tumor samples were digested in type V collagenase (MilliporeSigma) for 30 min prior to passing through the mesh. Cells were then incubated with antibodies conjugated with fluorochromes specific for CD3, CD4, CD8, CD11b, CD11c, CD206, PD-1, PD-L1, Ly6C, and Ly6G. Lymphocyte cell populations were also stained for intracellular TNF-α and IFN-γ production. Briefly, cells were stimulated with PMA and ionomycin (BioLegend, San Jose, CA, USA) for 6 h, then stained for intracellular TNF-α and IFN-γ. Samples were run using BD FACS Calibur or BD FACS Aria (BD Biosciences, San Jose, CA, USA) and analysis was performed using FlowJo software (Tree Star, Inc., Ashland, OR, USA).

### Real Time Quantitative PCR

Tumor, lymph node and spleen samples were stored in 500 μL of RNAlater (Thermofisher Scientific, Foster City, CA, USA) and frozen at −80°C for future analysis. Tissues were lysed and homogenized in TRIzol using a Bead Ruptor Elite (Omni International, Kennesaw, GA, USA). RNA was extracted using Direct-zol RNA Miniprep kit (Zymo Research, Irvine, CA, USA) and reverse transcribed to cDNA using the High Capacity cDNA Reverse Transcription Kit (Applied Biosystems, Foster City, CA, USA). Primer sequences for PCR were created using the IDT RealTime qPCR Tool (https://www.idtdna.com/scitools/Applications/RealTimePCR/, Integrated DNA Technologies, Coralville, IA, USA) (26, 27). Real time PCR of cDNA samples was performed using the PowerUp SYBR Green Master Mix (Thermofisher Scientific, Foster City, CA, USA) with beta actin (*Actb*) as a reference gene. Gene transcripts amplified include *VegfA, Hif1*α*, Prf1, Tnf, Ifng, Rorgc, Il1b, Il17, Pdl1, Klrg1, Klrc1, and Arg1*.

### T Cell Stimulation and ELISA

Single cell suspensions generated from spleen and lymph node tissues were incubated in the presence of αCD3 and αCD28 antibodies (Biolegend, San Diego, CA, USA) for 72 h. Cell supernatants pulled from stimulated and non-stimulated cell samples were analyzed for TNF-α and IFN-γ production. Sandwich ELISA was used to determine cytokine concentrations. Capture and detection antibodies for ELISA were purchased from Biolegend (San Diego, CA, USA).

### Histopathology and Immunohistochemistry

Primary tumors, draining cervical lymph nodes and spleens were fixed in 10% neutral buffered formalin followed by paraffin embedding. Paraffinized tissue sections 5 μm thick were cut for histopathology and immunohistochemistry. Histopathological samples were stained with hematoxylin and eosin. For immunohistochemistry, tissue sections were rehydrated in xylenes and graded ethanol before heat induced epitope retrieval. After antigen retrieval, IHC samples were blocked for endogenous peroxidase activity in 0.3% hydrogen peroxide in methanol for 30 minutes. Tissue sections were blocked for 30 min in 10% normal goat serum prior to overnight incubation with rat monoclonal anti-mouse Gr-1 primary antibody (BD Biosciences, San Jose, CA). Samples were incubated for 1 h with HRP conjugated goat polyclonal anti-rat IgG secondary antibody (Southern Biotech, Birmingham, AL, USA). DAB peroxidase kit (Vector Laboratories Inc., Burlingame CA) was used to detect for the presence of HRP. For immunofluorescence staining, hamster anti-mouse CD3 primary antibody (BD Biosciences, San Jose, CA) was used at a concentration of 0.625 μg/mL. Samples were incubated with Alexa Fluor 488 conjugated goat anti hamster secondary antibody (Thermofisher Scientific, Foster City, CA, USA) at a concentration of 2 μg/mL for 1 h, then counterstained with DAPI (BioLegend, San Diego, CA). Confocal imaging was performed using a Zeiss LSM 700 confocal microscope and analyzed with ZEN imaging software (Carl Zeiss, Munich, Germany).

### Statistical Analysis

Statistical analysis of samples was performed using GraphPad Prism v8.0.2 (GraphPad Software, San Diego, CA, USA). Student's *T*-test was used to determine statistically significant differences. Significance was determined by *p*-value threshold of 0.05.

## Results

### STAT4 Mediated Immune Pathways Are Important in Preventing Metastatic Progression in HNSCC

**Figure 1:**
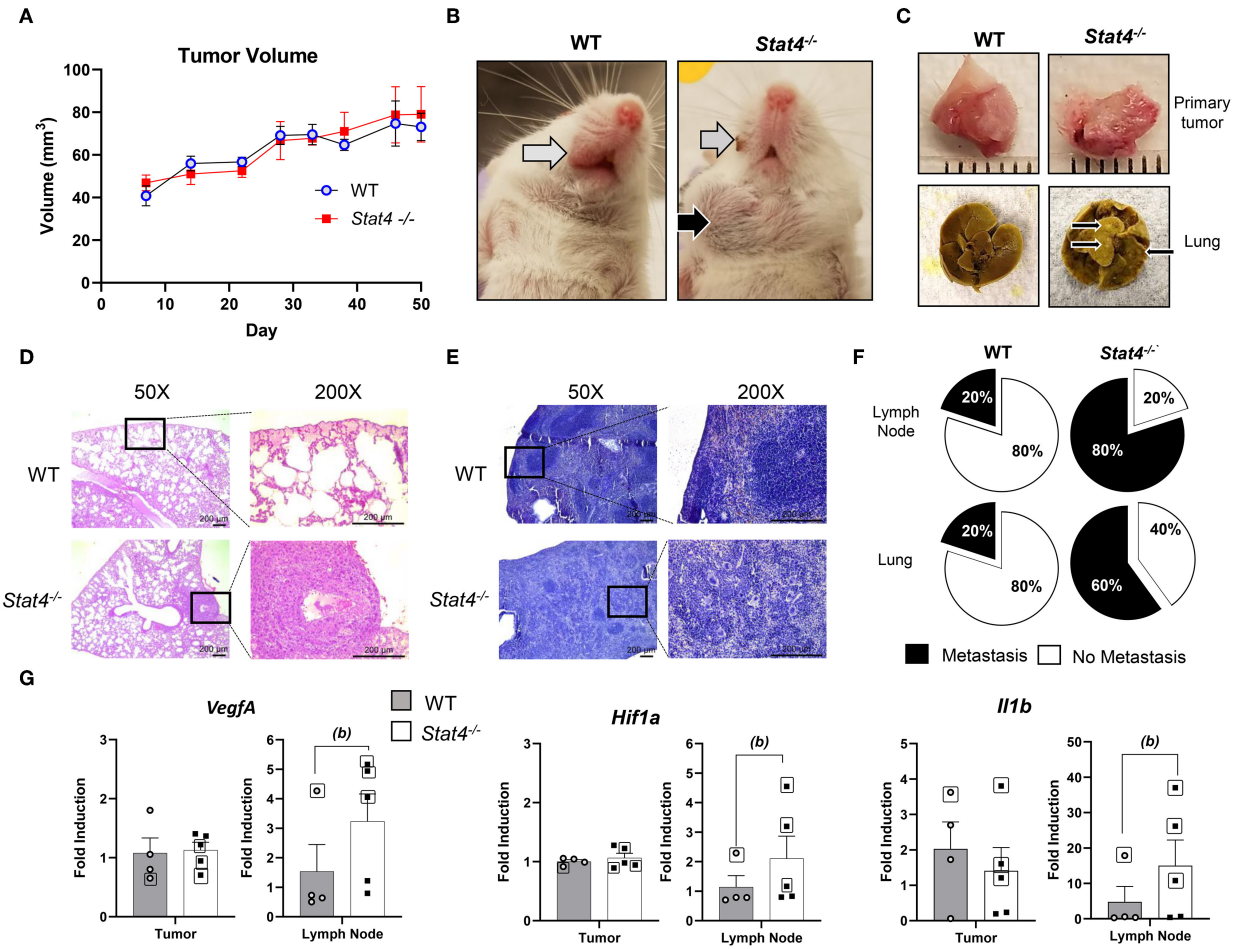
STAT4 mediated immune pathways are important in preventing metastatic progression in HNSCC. **(A)** Tumor volumes in tumor bearing WT (*n* = 5) and *Stat4*^−/−^ (*n* = 5) mice. **(B)** Representative images of the anterior cervical region of experimental mice in tumor bearing WT mouse lacking lymphatic metastases (left) and *Stat4*^−/−^ mouse bearing prominent lymphatic metastases (right). Primary tumor sites are indicated by the gray arrows while lymph node metastasis is indicated by the black arrow. **(C)** Representative images of primary tumors and lungs excised from a WT and a *Stat4*^−/−^ mice. Images were acquired at 50 days post HNSCC injection. Metastatic nodules on the lungs are indicated by the black arrows. **(D)** Representative histological images of lung sections from tumor bearing WT and *Stat4*^−/−^ mice stained with hematoxylin and eosin at X50 and X200 magnifications. Metastatic LY2 tumor cells in *Stat4*^−/−^ lung tissue is clearly shown in the black box. Scale bar represents 200 μm. **(E)** Representative histological images of lymph node sections from tumor bearing WT and *Stat4*^−/−^ mice stained with hematoxylin and eosin at X50 and X200 magnifications. Metastatic regions in *Stat4*^−/−^ lymph nodes are shown. Scale bar represents 200 μm. **(F)** Pie chart showing rates of lymph node and lung metastasis between tumor bearing *Stat4*^−/−^and WT mice (*n* = 5 per group). **(G)** Gene expression of *Vegfa, Hif1a, and Il1b* at primary tumor sites and sentinel lymph nodes of tumor bearing WT and *Stat4*^−/−^ mice as determined by RT-qPCR. Boxed data points are from mice showing lung metastases. (a) represents *p* < *0.05* for comparisons between tumor bearing WT and *Stat4*^−/−^ mice and (b) represents *p* < *0.05* for comparisons between tumor bearing WT mice and metastatic tumor bearing *Stat4*^−/−^ mice.

*Stat4*^−/−^ and WT mice (*n* = 5 per group) were monitored for a span of 50 days after orthotopic injection of LY2 cells. One *Stat4*^−/−^ was removed at day 42, having reached a moribund state associated with advanced tumor progression before terminal sacrifice. Primary tumor volumes were similar between tumor bearing *Stat4*^−/−^ and WT mice (Figures 1A–C). However, a significant portion of *Stat4*^−/−^ mice showed prominent cervical lymphadenopathy (Figure 1B) and metastatic nodules on their lungs (Figure 1C), both of which were mostly absent in WT mice. Histological analysis of the lungs and lymph nodes by a certified pathologist confirmed that metastasis to these sites occurred at a much higher rate in tumor bearing *Stat4*^−/−^ mice compared to tumor bearing WT mice (Figures 1D,E). Remarkably, we observed lymph node metastasis in 80% and lung metastasis in 60% of tumor bearing *Stat4*^−/−^ mice compared with WT who displayed incidences of only 20% for both draining lymph node and lung metastasis (Figure 1F). Representative images of cervical region containing the metastatic lymph nodes (Figure 1B), primary tumors and lungs (Figure 1C), and H&E stains of lung (Figure 1D) and lymph node tissue (Figure 1E) of WT and *Stat4*^−/−^ mice are shown. Taken together, our data suggests that although STAT4 deficiency does not affect primary tumor development, STAT4 associated pathways are important in preventing tumor metastasis in experimental HNSCC.

The increased rate of metastasis in tumor bearing *Stat4*^−/−^ mice led us to examine transcriptional expression of HNSCC biomarkers at the primary and metastatic tumor sites (lymph node). Specifically, we analyzed markers of angiogenesis (*VegfA*), hypoxia (*Hif1a*), and inflammation (*l1b*) (28). Interestingly, while there were no differences in gene expression of these biomarkers at the primary tumor site, significant increases were observed in the lymph nodes of mice with metastasis, indicating upregulated tumorigenic pathways at this site (Figure 1G). This was not surprising given the similar tumor volumes observed at the primary site of tumor bearing WT and *Stat4*^−/−^ mice (Figure 1A), in contrast to the increased lymph node metastatic sites seen in *Stat4*^−/−^ mice. Taken together, our data demonstrates increased metastatic progression in tumor bearing *Stat4*^−/−^ mice compared to tumor bearing WT mice.

### STAT4 Deficiency Promotes T Cell Immunosuppression and Diminishes Anti-tumoral Response to HNSCC

**Figure 2:**
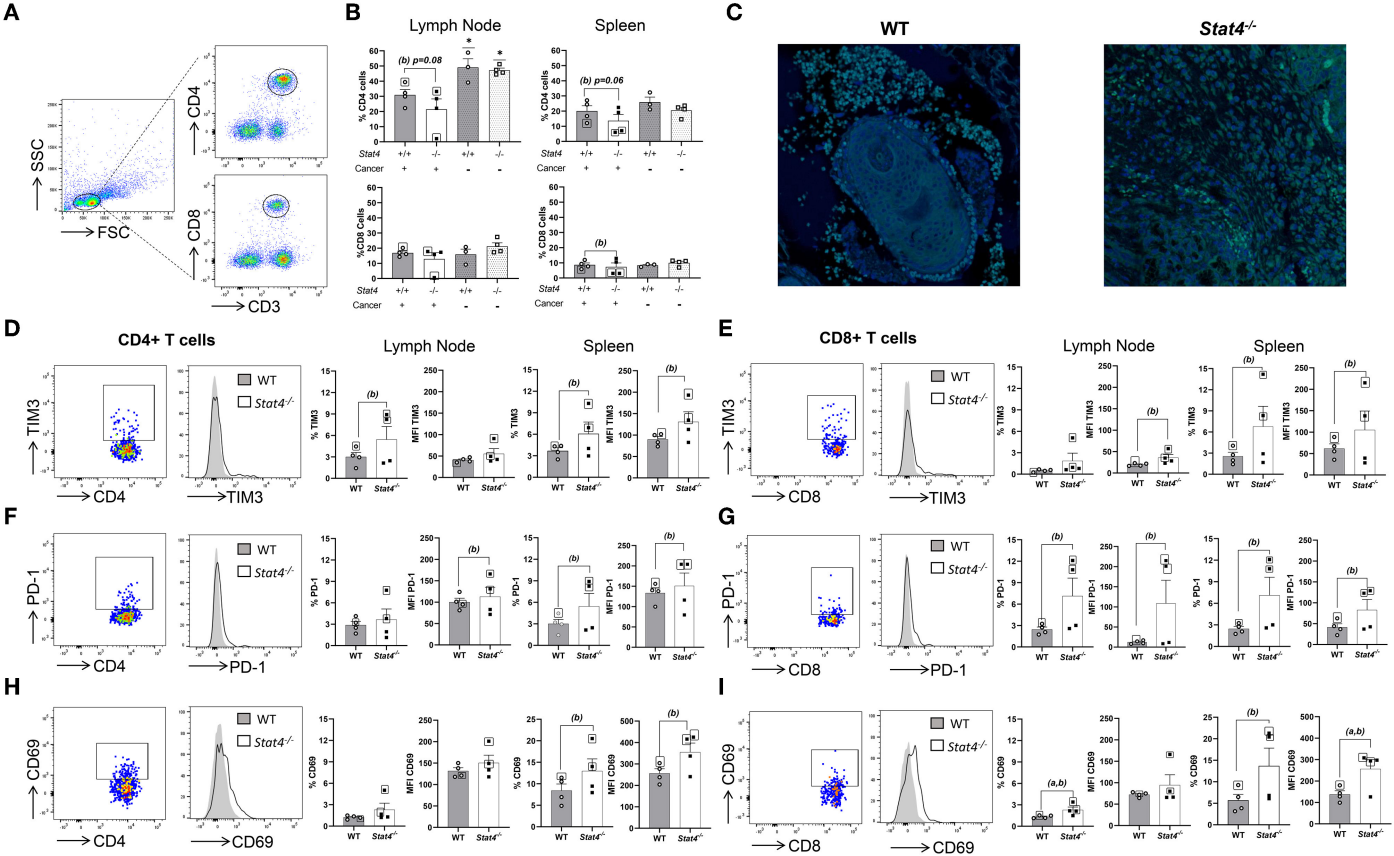
STAT4 deficiency promotes T cell immunosuppression and diminishes anti-tumoral response to HNSCC. **(A)** Gating strategy for determination of CD4^+^ and CD8^+^ T cell populations. **(B)** Percentage of CD4^+^ and CD8^+^ T cells in the draining lymph nodes and spleens of both tumor bearing and non tumor bearing WT and *Stat4*^−/−^ mice as determined by flow cytometric analysis. Significant differences between the tumor-bearing mice and non-tumor bearing mice of each mouse genotype are indicated by an * above the non-tumor bearing group plots. Data are presented as percentage of live cells. **(C)** Representative immunofluorescent images of CD3^+^ cells (green) in tumors of WT and *Stat4*^−/−^ mice. Tissues were counterstained with DAPI (blue). **(D–I)** Flow cytometric analysis showing intracellular expression of **(D,E)** TIM3, **(F,G)** PD-1, **(H,I)** CD69 by lymph node and splenic CD4^+^ and CD8^+^ populations of tumor bearing WT and *Stat4*^−/−^ mice. Representative dot plots and overlaid histogram plots of cellular expression of these immunosuppressive markers on gated CD4^+^ or CD8^+^ T cell populations are shown. Percentages of positive cells expressing these markers and mean fluorescent intensities (MFI) graphically represented to show differences between groups. Boxed data points are from mice showing lung metastases. (a) represents *p* < *0.05* for comparisons between tumor bearing WT and *Stat4*^−/−^ mice and (b) represents *p* < *0.05* for comparisons between tumor bearing WT mice and metastatic tumor bearing *Stat4*^−/−^ mice.

The significantly increased metastasis observed in tumor bearing *Stat4*^−/−^ mice relative to WT led us to examine the effect of STAT4 deficiency on anti-tumor T cell responses. We chose to focus on metastatic immunological sites and systemic immune responses, given that primary tumor development was similar between tumor bearing WT and *Stat4*^−/−^ mice. First, we observed significant reduction in CD4^+^ T cells in lymph nodes of all tumor bearing WT and *Stat4*^−/−^ mice compared to non-tumor bearing mice (Figures 2A,B). Further, while no significant differences were observed in draining lymph node T cell populations of tumor bearing WT and *Stat4*^−/−^ mice, reductions in splenic CD4^+^ T cell and CD8^+^ T cell populations were pronounced heavily in mice with lung metastases, particularly in metastatic *Stat4*^−/−^ mice (Figure 2B). Immunofluorescence staining of tumors of WT and *Stat4*^−/−^ mice confirmed T cell infiltration to oral tumor sites (Figure 2C).

Next, we evaluated the effect of STAT4 on T cell immunosuppressive functions, which are known to promote metastasis during HNSCC. TIM3, a potent T cell suppressor of the immune response (29), was found to be significantly overexpressed in the lymph node and splenic CD4^+^ and splenic CD8^+^ T cells of metastatic *Stat4*^−/−^ mice compared to tumor bearing WT mice (Figures 2D,E). Similarly, PD-1, a negative regulator of adaptive anti-tumor immunity (30, 31), was increased in lymph node and splenic CD8^+^ T cells and splenic CD4^+^ of metastatic *Stat4*^−/−^ mice compared to tumor bearing WT mice (Figures 2F,G). Increased expression of these immune checkpoint receptors in CD4+ and CD8+ T cells of HNSCC tumor bearing *Stat4*^−/−^ mice demonstrate the requirement for STAT4 in the inhibition of immunosuppressive T cell pathways and initiation of an appropriate immune response against metastatic HNSCC. Our flow cytometric analysis also revealed a substantial increase in CD69 expression in lymph node and splenic CD8^+^ T and splenic CD4^+^ lymphocytes of metastatic *Stat4*^−/−^ mice (Figures 2H,I). CD69 expression has recently been linked with exhaustion of cytotoxic T lymphocytes and NK cells (32). Interestingly, co-expression of PD1 and TIM3 has previously been found to be associated with exhaustion of CD8^+^ T cells during HNSCC (33) and colorectal cancer (34), and anti-TIM3 antibodies ameliorated these effects (29). Further, CD69 antibody blockade reduces PD-1 and TIM3 expression in CD8^+^ T cells, which is associated with reduced lung metastasis in breast cancer (32). Taken together, our results indicate that enhanced expression of T cell immunosuppressive biomarkers resulting in a diminished anti-tumor T-cell immune response in the absence of STAT4 potentially contributes to the increased HNSCC tumor metastasis observed in in *Stat4*^−/−^ mice.

### STAT4 Deficiency Promotes Accumulation of Immunosuppressive Myeloid Cells Systemically and at Metastatic Sites

**Figure 3:**
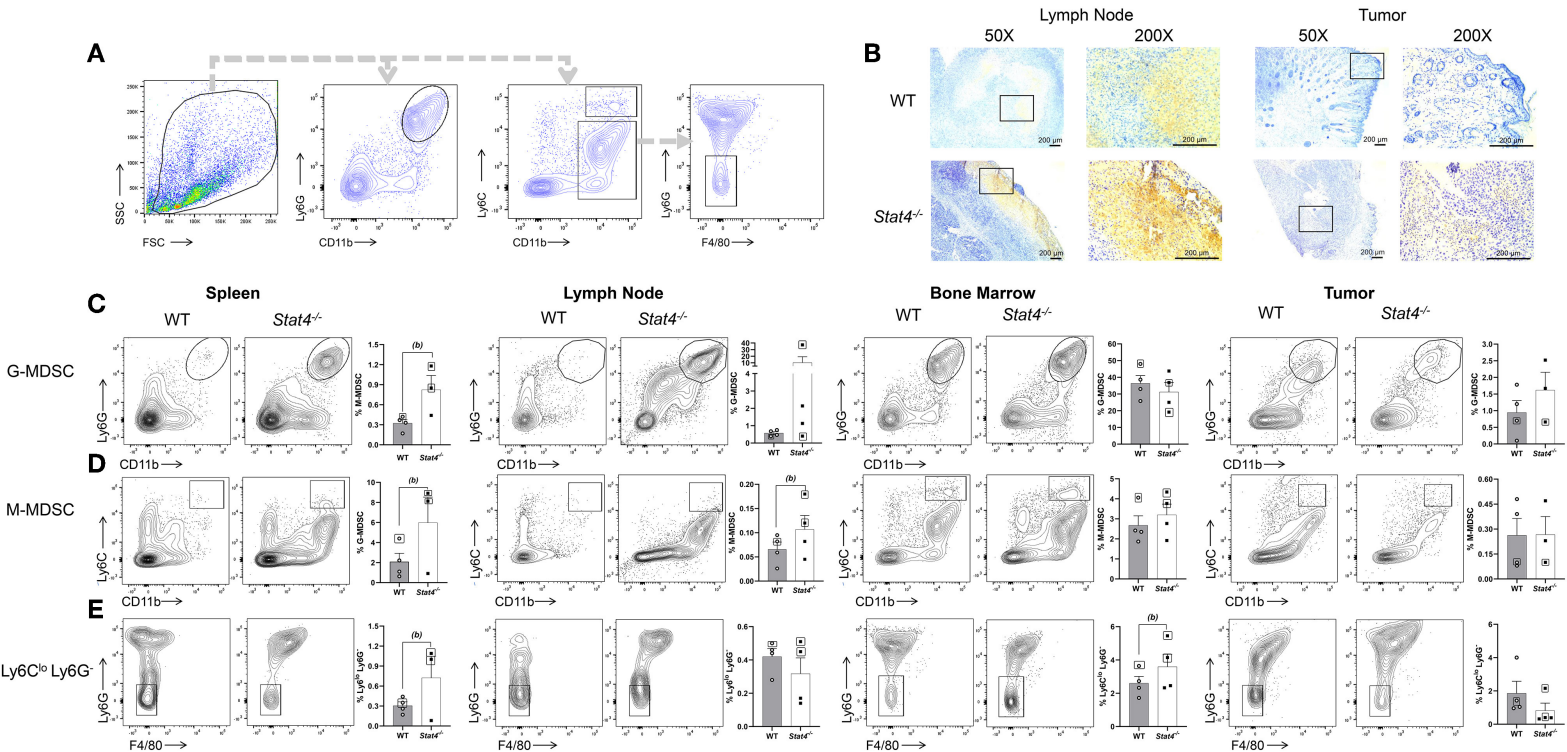
STAT4 deficiency promotes accumulation of immunosuppressive myeloid cells systemically and at metastatic sites. **(A)** General flow cytometric gating strategy used to define CD11b^+^Ly6G^hi^Ly6C^int^, CD11b^+^Ly6G^−^Ly6C^hi^, and CD11b^+^Ly6C^lo^Ly6G^−^ populations within all analyzed tissues. **(B)** Representative immunohistochemical images of lymph node and primary tumor sections from tumor bearing WT and *Stat4*^−/−^ mice stained with Gr-1 antibodies. **(C)** Representative flow cytometric plots and population distributions of CD11b^+^Ly6G^hi^Ly6C^int^ cells in the spleens, lymph nodes, bone marrow, and tumors of tumor bearing WT and *Stat4*^−/−^ mice. Data are presented as percentage of live cells. **(D)** Representative flow cytometric plots and population distributions of CD11b^+^Ly6G^−^Ly6C^hi^ cells in the spleens, lymph nodes, bone marrow, and tumors of tumor bearing WT and *Stat4*^−/−^ mice. Data are presented as percentage of live cells. **(E)** Representative flow cytometric plots and population distributions of CD11b^+^Ly6G^−^Ly6C^lo^ cells in the spleens, lymph nodes, and bone marrow of tumor bearing WT, and *Stat4*^−/−^ mice. Data are presented as percentage of live cells. Boxed data points are from mice showing lung metastases. (a) represents *p* < *0.05* for comparisons between tumor bearing WT and *Stat4*^−/−^ mice and (b) represents *p* < *0.05* for comparisons between tumor bearing WT mice and metastatic tumor bearing *Stat4*^−/−^ mice.

Myeloid cells in the tumor microenvironment affect HNSCC invasion and metastatic spread. CD11b^+^Ly6G^+^Ly6C^int^ and CD11b^+^Ly6C^hi^Ly6G^−^ myeloid cell populations, referred to as granulocytic and monocytic myeloid derived suppressor cells (G-MDSCs and M-MDSCs, respectively) are known to promote metastasis in HNSCC. We therefore investigated the effect of STAT4 deficiency on the accumulation and immunosuppressive potential of these myeloid cell populations during HNSCC by flow cytometry (Figure 3A) and immunohistochemistry (Figure 3B). Spleens and draining lymph nodes of tumor bearing *Stat4*^−/−^ mice were found to contain increased accumulation of CD11b^+^Ly6G^+^Ly6C^int^ and CD11b^+^Ly6G^−^Ly6C^hi^ cells compared to WT controls, particularly in metastatic mice (Figures 3C,D). Each of these myeloid cell populations are known to inhibit the ability of T lymphocytes to expand and exert their cytotoxic functions, suggesting a potential mechanism behind the impaired T cell anti-tumor responses observed in tumor bearing *Stat4*^−/−^ mice. Taken together, our data demonstrates a significant accumulation of MDSC populations in metastatic *Stat4*^−/−^ mice which potentially contributes to an immunosuppressive tumor microenvironment and subsequent metastasis in HNSCC. Interestingly, we observed that CD11b^+^Ly6C^lo^ Ly6G^−^ cells were more abundant in the spleen and bone marrow of tumor bearing *Stat4*^−/−^ mice compared to WT (Figure 3E). This population has previously been described to promote immunosuppression (35).

### Immunosuppressive G-MDSC Populations in Metastatic *Stat4^−/−^* Mice Display Significant Increases in Markers Associated With Tumor Progression

**Figure 4:**
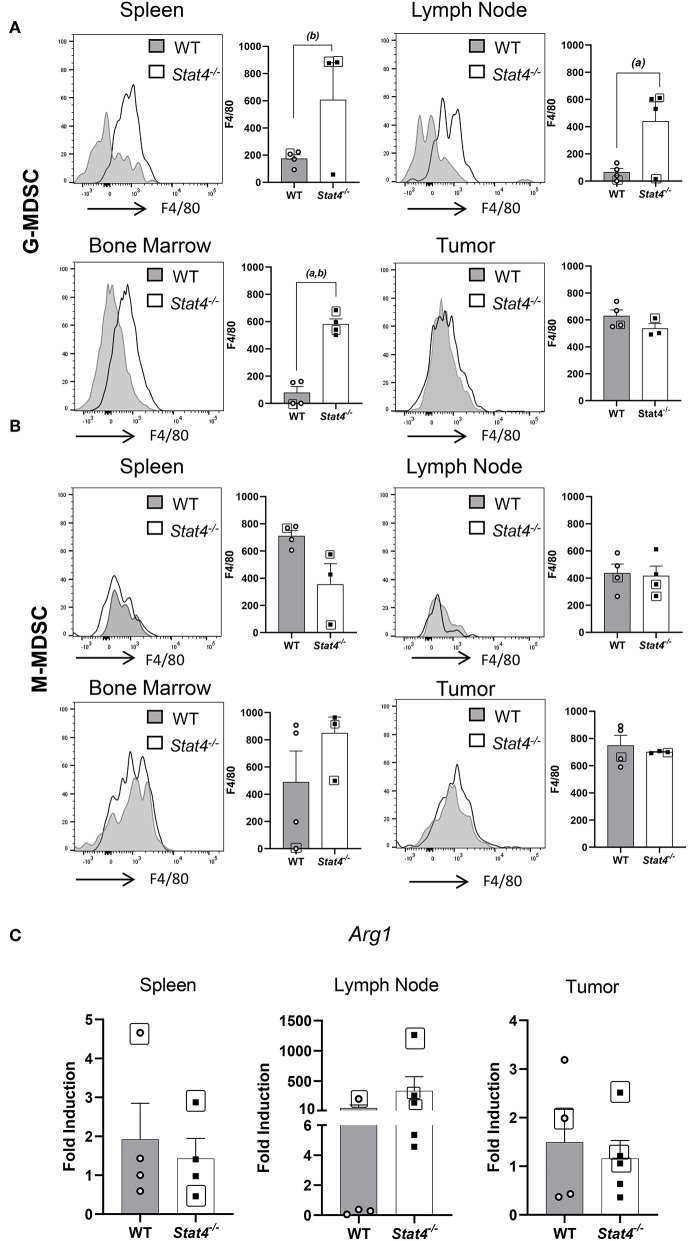
Immunosuppressive G-MDSC populations in metastatic *Stat4*^−/−^ mice display significant increases in markers associated with tumor progression. **(A,B)** Representative histogram plots showing F4/80 expression in **(A)** CD11b^+^Ly6G^hi^Ly6C^int^ G-MDSC cells and **(B)** CD11b^+^Ly6G^−^Ly6C^hi^ cells. MFIs of these cell populations in spleens, lymph nodes, tumors and bone marrow of tumor bearing WT and *Stat4*^−/−^ mice are graphically shown. **(C)** *Arg1* mRNA expression in sentinel lymph nodes, tumors, and spleens of tumor bearing WT and *Stat4*^−/−^ mice as determined by RT qPCR. Boxed data points are from mice showing lung metastases. (a) represents *p* <*0.05* for comparisons between tumor bearing WT and *Stat4*^−/−^ mice and (b) represents *p* <*0.05* for comparisons between tumor bearing WT mice and metastatic tumor bearing *Stat4*^−/−^ mice.

The observed increase in tumor-promoting myeloid populations in the spleens and lymph nodes of *Stat4*^−/−^ mice led us to determine their immunosuppressive potential. We analyzed the expression of surface markers associated with an immunosuppressive or pro-tumoral microenvironment, including F4/80, where we found significantly increased expression among G-MDSCs from *Stat4*^−/−^ mice. While G-MDSC populations expressing F4/80 have been identified in previous studies (36, 37), the function of this population remains unclear, although F4/80 has interestingly been associated with the induction of CD8^+^ Treg mediated peripheral tolerance (38). Flow cytometric analysis of F4/80 expression as measured through mean fluorescence intensity revealed that granulocytic CD11b^+^Ly6C^int^Ly6G^hi^ cells in the spleens and bone marrow of tumor bearing *Stat4*^−/−^ mice over-expressed this cell surface receptor (Figure 4A). F4/80 expression among CD11b^+^Ly6C^hi^ Ly6G^−^ cells was similar between tumor bearing WT and *Stat4*^−/−^ mice (Figure 4B). Finally, *Arg1*, involved in the immunosuppressive function of MDSC (39), was found to be highly elevated in the draining lymph nodes of tumor bearing metastatic and non-metastatic *Stat4*^−/−^ mice compared to tumor bearing WT counterparts. Interestingly, *Arg* expression in the primary tumor site and spleens were comparable between tumor bearing WT and *Stat4*^−/−^ *mice* (Figure 4C). These results demonstrate that STAT4 inhibits the differentiation and accumulation of immunosuppressive myeloid cells at HNSCC metastatic sites. Taken together, the significantly increased expansion of immunosuppressive myeloid populations in *Stat4*^−/−^ mice potentially promotes tumor metastasis in HNSCC.

### Effect of STAT4 Deficiency on Cytotoxic Lymphocyte Activity During Metastatic HNSCC

**Figure 5:**
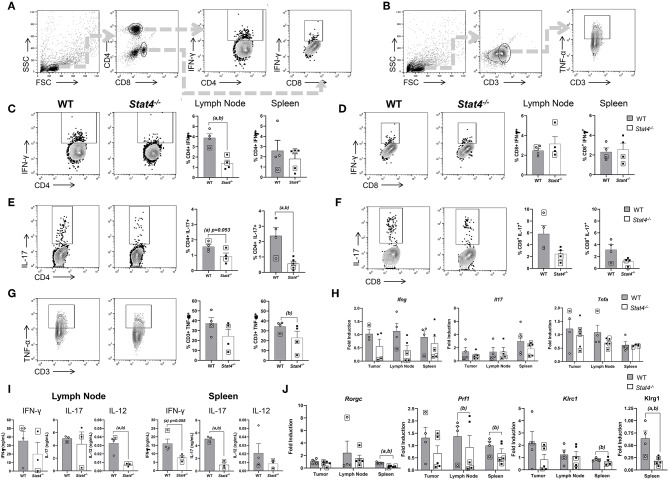
Effect of STAT4 deficiency on cytotoxic lymphocyte activity during metastatic HNSCC. **(A)** Flow cytometric gating strategy for determination of stimulated CD4^+^ and CD8^+^ T cell populations for analysis of IFN-γ and IL-17 expression. **(B)** Flow cytometric gating strategy for determination of stimulated CD3^+^ T cell populations for analysis of TNF-α expression. **(C,D)** Representative flow cytometric plots of IFN-γ production in CD4^+^ and CD8^+^ T cells of tumor bearing WT and *Stat4*^−/−^ mice along with their respective population distributions in lymph nodes and spleens of experimental mice. **(E,F)** Representative flow cytometric plots of IL-17 production in CD4^+^ and CD8^+^ T cells of tumor bearing WT and *Stat4*^−/−^ mice along with their respective population distributions in lymph nodes and spleens of experimental mice. **(G)** Representative flow cytometric plots of TNF-α production in CD3^+^ T cells of tumor bearing WT and *Stat4*^−/−^ mice along with their respective population distributions in lymph nodes (left) and spleens (right) of experimental mice. **(H)** *Ifng, Il-17*, and *Tnfa* gene expression measured by RT qPCR, derived from the tumors, sentinel lymph nodes and spleens of WT, and *Stat4*^−/−^ tumor bearing mice. **(I)** IFN-γ, IL-17, and IL-12 protein concentrations, quantified using ELISA, from the supernatants of CD3 restimulated cells isolated from the lymph nodes and spleens of tumor bearing mice. **(J)** *Rorgc, Prf1, Klrc1, and Klrg1* gene expression measured by RT-qPCR, using the tumors, sentinel lymph nodes and spleens of tumor bearing WT and *Stat4*^−/−^ mice. Boxed data points are from mice showing lung metastases. (a) represents *p* < *0.05* for comparisons between tumor bearing WT and *Stat4*^−/−^ mice and (b) represents *p* < *0.05* for comparisons between tumor bearing WT mice and metastatic tumor bearing *Stat4*^−/−^ mice.

Given the increased expression of immunosuppressive biomarkers observed in T cell and myeloid cell populations of HNSCC tumor-bearing *Stat4*^−/−^ mice, we determined the cytotoxic potential of lymphocyte populations involved in anti-tumor immune responses against HNSCC. To do this, spleen and lymph node cells of tumor bearing WT and *Stat4*^−/−^ mice were restimulated with PMA and ionomycin for 6 hours then analyzed for intracellular cytokine production (Figures 5A,B). Production of the anti-tumor cytokine IFN-γ, by CD4^+^ T cells was significantly attenuated in the draining lymph nodes of tumor bearing *Stat4*^−/−^ mice compared to WT (Figure 5C), although no differences were observed in CD8^+^ T cells (Figure 5D). Similar trends were found in *Ifng* gene expression in tumors, lymph nodes and spleens of tumor bearing *Stat4*^−/−^ mice, although not statistically significant (Figure 5H). Next, to confirm that *Stat4*^−/−^ T cells from HNSCC tumor bearing mice were deficient in their ability to produce IFN-γ, we re-stimulated splenocytes from tumor bearing WT and *Stat4*^−/−^ mice with anti-CD3 and anti-CD28 antibodies, then determined IFN-γ production by ELISA. As expected, restimulated splenic T cells from tumor bearing *Stat4*^−/−^ mice produced less IFN-γ than their WT counterparts (Figure 5I).

Next, we determined the effect of Stat4 deficiency on IL-17 production since T_H_17 development is partly mediated by STAT4 (40) and IL-17 production by T cells has been shown to play controversial roles in HNSCC (41, 42). We therefore analyzed IL-17 production in draining lymph nodes and spleens of tumor bearing WT and *Stat4*^−/−^ mice. Splenic CD4^+^ T cells of tumor bearing *Stat4*^−/−^ mice produced less IL-17 compared to WT mice. Similar results were observed in the draining lymph nodes of *Stat4*^−/−^ mice, although to a lesser extent (Figure 5E). Similar trends were observed in CD8 T cells although not statistically significant (Figure 5F). Our flow cytometry results correlated with gene expression of the T_H_17 transcription factor *Rorgc* in the spleens and lymph nodes, while tumor *Rorgc* expression were similar in WT and *Stat4*^−/−^ mice (Figure 5J). Similarly, CD3 re-stimulation of splenic T cells resulted in reduced expression of IL-17 in *Stat4*^−/−^ mice compared to WT as determined by ELISA (Figure 5I). Interestingly, IL-17 gene expression in the tumors, draining lymph nodes and spleens were similar between WT and *Stat4*^−/−^ tumor bearing mice, which may be due to IL-17 production by non-Th17 cells (Figure 5H). Taken together, our data suggests a deficiency in systemic STAT4-mediated T_H_17 activity, which is potentially associated with higher metastasis in *Stat4*^−/−^ mice.

TNF-α^+^ production by T cells in draining lymph nodes were comparable between WT and *Stat4*^−/−^ tumor bearing mice, while splenic T cell TNF-α production was slightly attenuated in *Stat4*^−/−^tumor bearing mice (Figures 5G,H). Diminished TNF-α production is suggestive of decreased anti-tumor activity in T cells (43), although T-cell TNF-α production does not appear to be mediated by STAT4 in our experimental HNSCC model. Splenic and lymph node T-cells stimulated with αCD3 antibodies showed a significant reduction of IL-12 production in tumor bearing *Stat4* deficient mice (Figure 5I). However, this observation was similar to non-tumor bearing control mice, suggesting that the inherent inability of *Stat4*^−/−^ mice to optimally produce IL-12 is HNSCC independent. However, given that serum IL-12 levels are significantly lower in metastatic cancer patients compared to non-metastatic patients (44), it is possible that the diminished IL-12 production potentially contributes to the increased metastasis observed in tumor bearing *Stat4*^−/−^ mice.

Finally, we examined the impact of STAT4 signaling on lymphocyte cytotoxic activity demonstrated by that the expression of perforin (*Prf1*), a cytolytic protein released by cytotoxic lymphocytes. We observed decreased *Prf1* expression in the draining lymph nodes and spleens of *Stat4*^−/−^ mice with lung metastases. Further, *Klrc1*, an NK cell marker, were decreased in the draining lymph nodes and spleens of *Stat4*^−/−^ mice with lung metastases, indicating diminished anti-tumor response by cytotoxic lymphocytes. Additionally, *Klrg1*, a marker associated with highly cytotoxic NK cell populations (45), was significantly decreased in the spleens of *Stat4*^−/−^ mice compared to WT (Figure 5J). Taken together, the reduction in cytotoxic lymphocyte activity observed systemically, as well as in metastatic but not primary tumor sites of *Stat4*^−/−^ mice, potentially contributes to metastasis in experimental HNSCC.

## Discussion

The results of our studies highlight the essential role for STAT4 in controlling HNSCC metastasis. The orthotopic murine experimental model of HNSCC using the metastatic HNSCC cell line, LY2, injected into immunocompetent BALB/c mice is a well-established model which has been used to study HNSCC metastasis. We showed that *Stat4*^−/−^ mice display a deficient anti-tumor immune response associated with higher rates of metastasis to both the local cervical lymph nodes and the lungs. Interestingly, primary tumor development and biomarkers associated with HNSCC tumor progression at the tumor site was comparable between WT and *Stat4*^−/−^ mice. This may be indicative of similar primary tumor site-specific immune responses. The primary difference between tumor bearing WT and *Stat4*^−/−^ mice lied in the inability of *Stat4*^−/−^ mice to prevent the establishment of tumors at metastatic sites (lymph nodes and lungs). Given that metastasis is a major cause of death among HNSCC patients, our results suggest that complementary strategies that enhance STAT4 activation might reduce HNSCC mortality. However, elucidation of the mechanisms that underlie STAT4 mediated inhibition of HNSCC metastasis would be needed to advance the application of STAT4 signaling in HNSCC treatment. Metastasis is a highly complex process (46), and our results are the first step toward understanding the potential cellular and molecular pathways involved in the reduction of HNSCC metastasis by STAT4.

In addition to mounting an effective immune response at the tumor site, our data highlights the importance of an appropriate anti-tumor immune response at the sentinel lymph node, in order to prevent the seeding and establishment of HNSCC cells at metastatic sites (47, 48). It is now well known that lymphatic metastasis is an active process, which is regulated at several steps. Therefore, a clear understanding of the lymph node microenvironment and the immunological factors governing lymphatic metastasis are essential to the effective design of therapeutic approaches that reduce mortality in HNSCC. Our studies clearly identify STAT4 as a major player in remodeling sentinel lymph nodes and regulating lymphatic metastasis during HNSCC.

A key process in sentinel lymph node metastasis is lymphangiogenesis, which favors tumor cell colonization and proliferation in sentinel lymph nodes. Indeed, a distinctive feature of pre-metastatic lymph nodes is increased lymphangiogenesis marked by the expression of *VegfA* (49, 50). Our data demonstrates that lymphangiogenesis is upregulated in tumor bearing *Stat4* deficient mice, as marked by increased *VegfA* expression in the lymph node, leading to an immune suppressive microenvironment that favors tumor growth. *Hif1a*, a transcription factor activated under hypoxic conditions that is conducive to angiogenesis, tumor growth, and metastasis (51), was also found to be overexpressed in the sentinel lymph nodes, further reflective of an impaired ability in *Stat4*^−/−^ mice to counteract pro-tumor changes at these sites. It is noteworthy that expression levels of *VegfA* and *Hif1a* are higher in metastatic lymph nodes of WT and *Stat4*^−/−^ mice compared to primary tumors and non-metastatic lymph nodes of tumor bearing mice. This suggests to us that the increased levels of these inflammatory mediators are a consequence of immunological responses to the presence of HNSCC cells at draining lymph node sites. Future studies will determine which immune cells in the lymph node are the major producers of these mediators. We suspect that the increased MDSC populations in tumor bearing *Stat4*^−/−^ mice are partly responsible for the increased levels of *VegfA* and *Hif1a*. Other studies corroborate the involvement of *VegfA* and *Hif1a* in tumor invasion and metastasis (52–54). Transcripts of *Il-1b*, a member of the IL-1 family which has been shown to be a prognostic for distant metastasis in HNSCC (28, 55, 56), was also significantly elevated in metastatic tumor bearing *Stat4*^−/−^mice. Many of the pro-tumorigenic effects of IL-1 are mediated by MDSC accumulation and angiogenesis (57, 58), which were also supported by our findings. All of these molecular changes observed in sentinel lymph nodes of *Stat4*^−/−^ tumor bearing mice support the crucial role STAT4 plays in the generation of a lymph node anti-tumor microenvironment unconducive to lymphatic metastasis and subsequent dissemination to distant organs during HNSCC (7).

Depending on their activation state and expression of tumor associated immunosuppressive markers, T lymphocytes play a major role in determining the outcome of HNSCC tumor development and metastasis (59–61). Tumor bearing *Stat4*^−/−^ mice showed trends toward decreases in CD4^+^ and CD8^+^ cells in lymph nodes and spleens, which was more pronounced in metastatic cases. Even more significant than a reduction in CD4^+^ and CD8^+^ T cell numbers in *Stat4*^−/−^ tumor bearing mice, was the strikingly elevated expression of the immunosuppressive markers PD-1 and TIM3 on splenic CD4^+^ and CD8^+^ T cells. TIM3 has been shown to induce T lymphocyte exhaustion, while TIM3 blockade promotes anti-tumor T cell immunity in HNSCC (29, 62–64). Similarly, TIM3 and PD-1 expression has been shown to correlate with pro-metastatic MDSC accumulation and immunosuppression of T cells in HNSCC (64, 65). Not surprisingly, *Stat4*^−/−^ mice displayed marked decreases in IFN-γ production by CD4^+^ cells of *Stat4*^−/−^ mice. However, IFN-γ production was not completely abrogated systemically, indicating that although not entirely STAT4 dependent, deficiency in IFN-γ production is partly mediated by STAT4 during metastatic HNSCC.

Given the importance of IL-17 on oral premalignant lesion development (41, 66), it was of interest to determine the impact of STAT4 inhibition on T_H_17 differentiation and effector mechanisms during metastatic HNSCC. Our results showed that STAT4 deficiency-induced systemic depletion of T_H_17 cells and IL-17 (indicated by lower CD4^+^ IL-17^+^ populations, and IL-17 expression and production), which correlated with increased HNSCC. However, total IL-17 expression at primary and metastatic tumor sites, as shown by IL-17 mRNA transcripts were similar for both WT and STAT4 deficient tumor bearing mice, suggesting that IL-17 expression by non-T cells, which is a potentially STAT4-independent process that may be playing a role. This is further supported by the significant attenuation of IL-17 production by T cell re-stimulated splenocytes and the diminished expression of *Rorgc* (a T_H_17 transcription factor) in the spleens of tumor bearing *Stat4*^−/−^ mice. Combined with the diminished IFN-γ production observed in tumor bearing *Stat4*^−/−^ mice, our data supports the hypothesis that STAT4-mediated induction of systemic T_H_1 and T_H_17 anti-tumor immune responses is essential for the inhibition of metastasis during HNSCC.

We analyzed the effect of STAT4 deficiency on immunosuppressive myeloid populations (MDSCs) during metastatic HNSCC. MDSCs are immature myeloid cells which have been shown to be potent mediators of immunosuppression in cancer, a significant factor in tumor evasion and distal metastasis in HNSCC (67). These cell populations are known to suppress the anti-tumor immune response, through inhibition of T lymphocyte expansion, differentiation and cancer cell cytotoxicity (68). Under inflammatory conditions, *Stat4*^−/−^ mice have been demonstrated to increase the accumulation of MDSCs (69). Consistent with these findings, our results showed that metastatic *Stat4*^−/−^ tumor mice displayed markedly large populations of CD11b^+^Ly6G^hi^Ly6C^int^ and CD11b^+^Ly6G^−^Ly6C^hi^ cells, representative of granulocytic and monocytic MDSCs respectively, in their lymph nodes and spleens, compared to WT. Given the previously established link between granulocytic CD11b^+^Ly6G^hi^Ly6C^int^ MDSC expansion and cancer metastasis (70, 71), our studies suggest a role for STAT4 in inhibiting immunosuppressive myeloid cell differentiation to prevent HNSCC establishment at lymph node metastatic sites.

Interestingly, we also observed a distinct population of CD11b^+^Ly6C^lo^Ly6G^−^ with increased accumulation in the spleens of tumor bearing *Stat4*^−/−^ mice compared to WT. Currently, the function of CD11b^+^Ly6C^lo^Ly6G^−^ cells is poorly understood, though they have been shown to be monocytic with immunosuppressive capability in a non-contact mediated manner by arginase and indoleamine 2,3-dioxygenase (72). Future studies will determine the exact role these cells play in tumor progression and metastasis, and the involvement of STAT4 in this process during HNSCC.

In summary, our results demonstrate that STAT4 is a key mediator in the inhibition of HNSCC tumor metastasis through mechanisms associated with increased T cell immunosuppression MDSC activity and pro-tumor inflammation, as well as a decrease in cytotoxic anti-tumor lymphocytic activity. This provides a strong rationale and opens the possibility of targeted immune based therapies that activate the STAT4 pathway in the treatment of metastatic malignancies. Future studies focused on potential causative factors underlying HNSCC tumor development and metastasis associated with STAT4 deficiency, as well as an expanded profile of the immune response elicited by metastatic tumor bearing *Stat4*^−/−^ mice will provide additional insights regarding targets of the STAT4 signaling pathway which can be explored in the treatment of lymphatic metastasis in HNSCC.

## Data Availability Statement

All datasets generated for this study are included in the article/supplementary material.

## Ethics Statement

The animal study was reviewed and approved by the Institutional Animal Care and Use Committee, The Ohio State University.

## Author Contributions

SO designed the study. KA, NR, GV, SV, and SO performed experiments and acquired data. KA, NR, GV, SV, AS, and SO analyzed and interpreted data. KA and SO drafted the manuscript. All authors critically revised and approved the final manuscript.

### Conflict of Interest

The authors declare that the research was conducted in the absence of any commercial or financial relationships that could be construed as a potential conflict of interest.

## References

[1] Ferlay J Steliarova-Foucher E Lortet-Tieulent J Rosso S Coebergh JW Comber H. Cancer incidence and mortality patterns in Europe: estimates for 40 countries in 2012. Eur J Cancer. (2013) 49:1374–403. 10.1016/j.ejca.2012.12.027 23485231

[2] Siegel RL Miller KD Jemal A Cancer statistics, 2017. CA Cancer J Clin. (2017) 67:7–30. 10.3322/caac.21387 28055103

[3] Rivera C. Essentials of oral cancer. Int J Clin Exp Pathol. (2015) 8:11884–94. 26617944 PMC4637760

[4] Nathanson SD Shah R Rosso K. Sentinel lymph node metastases in cancer: causes, detection and their role in disease progression. Semin Cell Dev Biol. (2015) 38:106–16. 10.1016/j.semcdb.2014.10.002 25444847

[5] Achen MG Stacker SA. Exit stage left: a tumor cell's journey from lymph node to beyond. Trends Cancer. (2018) 4:519–22. 10.1016/j.trecan.2018.05.007 30064660

[6] Nathanson SD Kwon D Kapke A Alford SH Chitale D. The role of lymph node metastasis in the systemic dissemination of breast cancer. Ann Surg Oncol. (2009) 16:3396–405. 10.1245/s10434-009-0659-2 19657697

[7] Pereira ER Kedrin D Seano G Gautier O Meijer EFJ Jones D. Lymph node metastases can invade local blood vessels, exit the node, and colonize distant organs in mice. Science. (2018) 359:1403–7. 10.1126/science.aal3622 29567713 PMC6002772

[8] Bowman T Garcia R Turkson J Jove R. STATs in oncogenesis. Oncogene. (2000) 19:2474–88. 10.1038/sj.onc.1203527 10851046

[9] Showe LC Fox FE Williams D Au K Niu Z Rook AH. Depressed IL-12-mediated signal transduction in T cells from patients with Sezary syndrome is associated with the absence of IL-12 receptor beta 2 mRNA and highly reduced levels of STAT4. J Immunol. (1999) 163:4073–9. 10491012

[10] Oppmann B Lesley R Blom B Timans JC Xu Y Hunte B. Novel p19 protein engages IL-12p40 to form a cytokine, IL-23, with biological activities similar as well as distinct from IL-12. Immunity. (2000) 13:715–25. 10.1016/S1074-7613(00)00070-4 11114383

[11] Townsend MJ Weinmann AS Matsuda JL Salomon R Farnham PJ Biron CA. T-bet regulates the terminal maturation and homeostasis of NK and Valpha14i NKT cells. Immunity. (2004) 20:477–94. 10.1016/S1074-7613(04)00076-7 15084276

[12] Kaplan MH Sun YL Hoey T Grusby MJ. Impaired IL-12 responses and enhanced development of Th2 cells in Stat4-deficient mice. Nature. (1996) 382:174–7. 10.1038/382174a0 8700209

[13] Varikuti S Oghumu S Natarajan G Kimble J Sperling RH Moretti E STAT4 is required for the generation of Th1 and Th2, but not Th17 immune responses during monophosphoryl lipid A adjuvant activity. Int Immunol. (2016) 28:565–70. 10.1093/intimm/dxw038 27578456 PMC6018885

[14] Thierfelder WE Van Deursen JM Yamamoto K Tripp RA Sarawar SR Carson RT. Requirement for Stat4 in interleukin-12-mediated responses of natural killer and T cells. Nature. (1996) 382:171–4. 10.1038/382171a0 8700208

[15] Kuroda E Kito T Yamashita U. Reduced expression of STAT4 and IFN-gamma in macrophages from BALB/c mice. J Immunol. (2002) 168:5477–82. 10.4049/jimmunol.168.11.5477 12023341

[16] Wang G Chen JH Qiang Y Wang DZ Chen Z. Decreased STAT4 indicates poor prognosis and enhanced cell proliferation in hepatocellular carcinoma. World J Gastroenterol. (2015) 21:3983–93. 10.3748/wjg.v21.i13.3983 25852285 PMC4385547

[17] Nishi M Batsaikhan BE Yoshikawa K Higashijima J Tokunaga T Takasu C. High STAT4 expression indicates better disease-free survival in patients with gastric cancer. Anticancer Res. (2017) 37:6723–9. 10.21873/anticanres.12131 29187449

[18] Zhang Y Yu C. Prognostic values of signal transducers activators of transcription in gastric cancer. Biosci Rep. (2019) 39. 10.1042/BSR20181695 30944204 PMC6488950

[19] Li S Sheng B Zhao M Shen Q Zhu H Zhu X. The prognostic values of signal transducers activators of transcription family in ovarian cancer. Biosci Rep. (2017) 37:BSR20181695. 10.1042/BSR20170650 28536310 PMC5518537

[20] Wang S Yu L Shi W Li X Yu L. Prognostic roles of signal transducers and activators of transcription family in human breast cancer. Biosci Rep. (2018) 38:BSR20171175. 10.1042/BSR20171175 29326301 PMC6294627

[21] Mirjačić Martinović K Babović N DŽodić R Jurišić V Matković S Konjević G Favorable *in vitro* effects of combined IL-12 and IL-18 treatment on NK cell cytotoxicity and CD25 receptor expression in metastatic melanoma patients. J Transl Med. (2015) 13:120 10.1186/s12967-015-0479-z 25889680 PMC4421987

[22] Mirjacic Martinovic K Srdic-Rajic T Babovic N Dzodic R Jurisic V Konjevic G. Decreased expression of pSTAT, IRF-1 and DAP10 signalling molecules in peripheral blood lymphocytes of patients with metastatic melanoma. J Clin Pathol. (2016) 69:300–6. 10.1136/jclinpath-2015-203107 26442832

[23] Zhao L Ji G Le X Luo Z Wang C Feng M. An integrated analysis identifies STAT4 as a key regulator of ovarian cancer metastasis. Oncogene. (2017) 36:3384–96. 10.1038/onc.2016.487 28114283

[24] Cheng JM Yao MR Zhu Q Wu XY Zhou J Tan WL. Silencing of stat4 gene inhibits cell proliferation and invasion of colorectal cancer cells. J Biol Regul Homeost Agents. (2015) 29:85–92. 25864744

[25] Vigneswaran N Wu J Song A Annapragada A Zacharias W. Hypoxia-induced autophagic response is associated with aggressive phenotype and elevated incidence of metastasis in orthotopic immunocompetent murine models of head and neck squamous cell carcinomas (HNSCC). Exp Mol Pathol. (2011) 90:215–25. 10.1016/j.yexmp.2010.11.011 21236253 PMC3057178

[26] Oghumu S Casto BC Ahn-Jarvis J Weghorst LC Maloney J Geuy P. Inhibition of pro-inflammatory and anti-apoptotic biomarkers during experimental oral cancer chemoprevention by dietary black raspberries. Front Immunol. (2017) 8:1325. 10.3389/fimmu.2017.01325 29109723 PMC5660285

[27] Varikuti S Oghumu S Elbaz M Volpedo G Ahirwar DK Alarcon PC. STAT1 gene deficient mice develop accelerated breast cancer growth and metastasis which is reduced by IL-17 blockade. Oncoimmunology. (2017) 6:e1361088. 10.1080/2162402X.2017.1361088 29147627 PMC5674966

[28] Lewis AM Varghese S Xu H Alexander HR. Interleukin-1 and cancer progression: the emerging role of interleukin-1 receptor antagonist as a novel therapeutic agent in cancer treatment. J Transl Med. (2006) 4:48. 10.1186/1479-5876-4-48 17096856 PMC1660548

[29] Liu JF Ma SR Mao L Bu LL Yu GT Li YC. T-cell immunoglobulin mucin 3 blockade drives an antitumor immune response in head and neck cancer. Mol Oncol. (2017) 11:235–47. 10.1002/1878-0261.12029 28102051 PMC5527458

[30] Nishimura H Nose M Hiai H Minato N Honjo T. Development of lupus-like autoimmune diseases by disruption of the PD-1 gene encoding an ITIM motif-carrying immunoreceptor. Immunity. (1999) 11:141–51. 10.1016/S1074-7613(00)80089-8 10485649

[31] Seidel JA Otsuka A Kabashima K. Anti-PD-1 and Anti-CTLA-4 therapies in cancer: mechanisms of action, efficacy, and limitations. Front Oncol. (2018) 8:86. 10.3389/fonc.2018.00086 29644214 PMC5883082

[32] Mita Y Kimura MY Hayashizaki K Koyama-Nasu R Ito T Motohashi S. Crucial role of CD69 in anti-tumor immunity through regulating the exhaustion of tumor-infiltrating T cells. Int Immunol. (2018) 30:559–67. 10.1093/intimm/dxy050 30085193

[33] Jie HB Srivastava RM Argiris A Bauman JE Kane LP Ferris RL. Increased PD-1(+) and TIM-3(+) TILs during cetuximab therapy inversely correlate with response in head and neck cancer patients. Cancer Immunol Res. (2017) 5:408–16. 10.1158/2326-6066.CIR-16-0333 28408386 PMC5497750

[34] Liu J Zhang S Hu Y Yang Z Li J Liu X. Targeting PD-1 and Tim-3 pathways to reverse CD8 T-cell exhaustion and enhance *ex vivo* T-cell responses to autologous dendritic/tumor vaccines. J Immunother. (2016) 39:171–80. 10.1097/CJI.0000000000000122 27070448

[35] Jung K Heishi T Khan OF Kowalski PS Incio J Rahbari NN. Ly6Clo monocytes drive immunosuppression and confer resistance to anti-VEGFR2 cancer therapy. J Clin Invest. (2017) 127:3039–51. 10.1172/JCI93182 28691930 PMC5531423

[36] Youn JI Nagaraj S Collazo M Gabrilovich DI. Subsets of myeloid-derived suppressor cells in tumor-bearing mice. J Immunol. (2008) 181:5791–802. 10.4049/jimmunol.181.8.5791 18832739 PMC2575748

[37] Damuzzo V Pinton L Desantis G Solito S Marigo I Bronte V. Complexity and challenges in defining myeloid-derived suppressor cells. Cytometry B Clin Cytom. (2015) 88:77–91. 10.1002/cytob.21206 25504825 PMC4405078

[38] Lin HH Faunce DE Stacey M Terajewicz A Nakamura T Zhang-Hoover J. The macrophage F4/80 receptor is required for the induction of antigen-specific efferent regulatory T cells in peripheral tolerance. J Exp Med. (2005) 201:1615–25. 10.1084/jem.20042307 15883173 PMC2212925

[39] Miret JJ Kirschmeier P Koyama S Zhu M Li YY Naito Y. Suppression of myeloid cell arginase activity leads to therapeutic response in a NSCLC mouse model by activating anti-tumor immunity. J Immunother Cancer. (2019) 7:32. 10.1186/s40425-019-0504-5 30728077 PMC6366094

[40] Mathur AN Chang HC Zisoulis DG Stritesky GL Yu Q O'malley JT. Stat3 and Stat4 direct development of IL-17-secreting Th cells. J Immunol. (2007) 178:4901–7. 10.4049/jimmunol.178.8.4901 17404271

[41] Qian X Chen H Wu X Hu L Huang Q Jin Y. Interleukin-17 acts as double-edged sword in anti-tumor immunity and tumorigenesis. Cytokine. (2017) 89:34–44. 10.1016/j.cyto.2015.09.011 26883678

[42] Lee MH Tung-Chieh Chang J Liao CT Chen YS Kuo ML Shen CR. Interleukin 17 and peripheral IL-17-expressing T cells are negatively correlated with the overall survival of head and neck cancer patients. Oncotarget. (2018) 9:9825–37. 10.18632/oncotarget.23934 29515773 PMC5839404

[43] Calzascia T Pellegrini M Hall H Sabbagh L Ono N Elford AR TNF-alpha is critical for antitumor but not antiviral T cell immunity in mice. J Clin Invest. (2007) 117:3833–45. 10.1172/JCI32567 17992258 PMC2066188

[44] Sparano A Lathers DM Achille N Petruzzelli GJ Young MR. Modulation of Th1 and Th2 cytokine profiles and their association with advanced head and neck squamous cell carcinoma. Otolaryngol Head Neck Surg. (2004) 131:573–6. 10.1016/j.otohns.2004.03.016 15523428

[45] Greenberg SA Kong SW Thompson E Gulla SV. Co-inhibitory T cell receptor KLRG1: human cancer expression and efficacy of neutralization in murine cancer models. Oncotarget. (2019) 10:1399–406. 10.18632/oncotarget.26659 30858925 PMC6402715

[46] Valastyan S Weinberg RA. Tumor metastasis: molecular insights and evolving paradigms. Cell. (2011) 147:275–92. 10.1016/j.cell.2011.09.024 22000009 PMC3261217

[47] Oghumu S Knobloch TJ Terrazas C Varikuti S Ahn-Jarvis J Bollinger CE. Deletion of macrophage migration inhibitory factor inhibits murine oral carcinogenesis: potential role for chronic pro-inflammatory immune mediators. Int J Cancer. (2016) 139:1379–90. 10.1002/ijc.30177 27164411 PMC4939094

[48] Ryan N Anderson K Volpedo G Hamza O Varikuti S Satoskar AR STAT1 inhibits T cell exhaustion and myeloid derived suppressor cell accumulation to promote anti-tumor immune responses in head and neck squamous cell carcinoma. Int J Cancer. (2019). 10.1002/ijc.32781. [Epub ahead of print]. PMC7366342 31709529

[49] Harrell MI Iritani BM Ruddell A. Tumor-induced sentinel lymph node lymphangiogenesis and increased lymph flow precede melanoma metastasis. Am J Pathol. (2007) 170:774–86. 10.2353/ajpath.2007.060761 17255343 PMC1851877

[50] Bielenberg DR Zetter BR. The contribution of angiogenesis to the process of metastasis. Cancer J. (2015) 21:267–73. 10.1097/PPO.0000000000000138 26222078 PMC4670555

[51] Muz B De La Puente P Azab F Azab AK. The role of hypoxia in cancer progression, angiogenesis, metastasis, and resistance to therapy. Hypoxia. (2015) 3:83–92. 10.2147/HP.S93413 27774485 PMC5045092

[52] Hirakawa S Kodama S Kunstfeld R Kajiya K Brown LF Detmar M. VEGF-A induces tumor and sentinel lymph node lymphangiogenesis and promotes lymphatic metastasis. J Exp Med. (2005) 201:1089–99. 10.1084/jem.20041896 15809353 PMC2213132

[53] Hu Z Fan C Livasy C He X Oh DS Ewend MG. A compact VEGF signature associated with distant metastases and poor outcomes. BMC Med. (2009) 7:9. 10.1186/1741-7015-7-9 19291283 PMC2671523

[54] Jing SW Wang YD Kuroda M Su JW Sun GG Liu Q. HIF-1α contributes to hypoxia-induced invasion and metastasis of esophageal carcinoma via inhibiting E-cadherin and promoting MMP-2 expression. Acta Med Okayama. (2012) 66:399–407. 10.18926/AMO/48964 23093058

[55] Voronov E Shouval DS Krelin Y Cagnano E Benharroch D Iwakura Y. IL-1 is required for tumor invasiveness and angiogenesis. Proc Natl Acad Sci USA. (2003) 100:2645–50. 10.1073/pnas.0437939100 12598651 PMC151394

[56] León X Bothe C García J Parreño M Alcolea S Quer M. Expression of IL-1α correlates with distant metastasis in patients with head and neck squamous cell carcinoma. Oncotarget. (2015) 6:37398–409. 10.18632/oncotarget.6054 26460957 PMC4741937

[57] Mantovani A Barajon I Garlanda C. IL-1 and IL-1 regulatory pathways in cancer progression and therapy. Immunol Rev. (2018) 281:57–61. 10.1111/imr.12614 29247996 PMC5922413

[58] Kaneko N Kurata M Yamamoto T Morikawa S Masumoto J. The role of interleukin-1 in general pathology. Inflamm Regen. (2019) 39:12. 10.1186/s41232-019-0101-5 31182982 PMC6551897

[59] Hodi FS Dranoff G. The biologic importance of tumor-infiltrating lymphocytes. J Cutan Pathol. (2010) 37 (Suppl. 1):48–53. 10.1111/j.1600-0560.2010.01506.x 20482675 PMC3905324

[60] Ruffell B Denardo DG Affara NI Coussens LM. Lymphocytes in cancer development: polarization towards pro-tumor immunity. Cytokine Growth Factor Rev. (2010) 21:3–10. 10.1016/j.cytogfr.2009.11.002 20005150 PMC2834837

[61] Hadrup S Donia M Thor Straten P. Effector CD4 and CD8 T cells and their role in the tumor microenvironment. Cancer Microenviron. (2013) 6:123–33. 10.1007/s12307-012-0127-6 23242673 PMC3717059

[62] Zhu C Anderson AC Schubart A Xiong H Imitola J Khoury SJ. The Tim-3 ligand galectin-9 negatively regulates T helper type 1 immunity. Nat Immunol. (2005) 6:1245–52. 10.1038/ni1271 16286920

[63] Ngiow SF Von Scheidt B Akiba H Yagita H Teng MW Smyth MJ. Anti-TIM3 antibody promotes T cell IFN-gamma-mediated antitumor immunity and suppresses established tumors. Cancer Res. (2011) 71:3540–51. 10.1158/0008-5472.CAN-11-0096 21430066

[64] Das M Zhu C Kuchroo VK. Tim-3 and its role in regulating anti-tumor immunity. Immunol Rev. (2017) 276:97–111. 10.1111/imr.12520 28258697 PMC5512889

[65] Guigay J Saada-Bouzid E Peyrade F Michel C. Approach to the patient with recurrent/metastatic disease. Curr Treat Options Oncol. (2019) 20:65. 10.1007/s11864-019-0664-z 31240480

[66] Caughron B Yang Y Young MRI. Role of IL-23 signaling in the progression of premalignant oral lesions to cancer. PLoS ONE. (2018) 13:e0196034. 10.1371/journal.pone.0196034 29664967 PMC5903614

[67] Umansky V Blattner C Gebhardt C Utikal J. The role of myeloid-derived suppressor cells (MDSC) in cancer progression. Vaccines. (2016) 4:36. 10.3390/vaccines4040036 27827871 PMC5192356

[68] Kumar V Patel S Tcyganov E Gabrilovich DI. The nature of myeloid-derived suppressor cells in the tumor microenvironment. Trends Immunol. (2016) 37:208–20. 10.1016/j.it.2016.01.004 26858199 PMC4775398

[69] Fu C Jiang L Xu X Zhu F Zhang S Wu X. STAT4 knockout protects LPS-induced lung injury by increasing of MDSC and promotingof macrophage differentiation. Respir Physiol Neurobiol. (2016) 223:16–22. 10.1016/j.resp.2015.11.016 26644077

[70] Cools-Lartigue J Spicer J Mcdonald B Gowing S Chow S Giannias B Neutrophil extracellular traps sequester circulating tumor cells and promote metastasis. J Clin Invest. (2013) 123:3446–58. 10.1172/JCI67484 PMC3726160 23863628

[71] Mcdonald B Spicer J Giannais B Fallavollita L Brodt P Ferri LE. Systemic inflammation increases cancer cell adhesion to hepatic sinusoids by neutrophil mediated mechanisms. Int J Cancer. (2009) 125:1298–305. 10.1002/ijc.24409 19431213

[72] Mckee SJ Tuong ZK Kobayashi T Doff BL Soon MS Nissen M. B cell lymphoma progression promotes the accumulation of circulating Ly6Clo monocytes with immunosuppressive activity. Oncoimmunology. (2018) 7:e1393599. 10.1080/2162402X.2017.1393599 29308328 PMC5749670

